# Detection of *Chlamydia* Developmental Forms and Secreted Effectors by Expansion Microscopy

**DOI:** 10.3389/fcimb.2019.00276

**Published:** 2019-08-09

**Authors:** Tobias C. Kunz, Ralph Götz, Markus Sauer, Thomas Rudel

**Affiliations:** ^1^Department of Microbiology, Julius-Maximilians-Universität Würzburg, Würzburg, Germany; ^2^Department of Biotechnology and Biophysics, Julius-Maximilians-Universität Würzburg, Würzburg, Germany

**Keywords:** expansion microscopy, chlamydia, secreted effectors, developmental forms, superresolution, imaging

## Abstract

Expansion microscopy (ExM) is a novel tool to improve the resolution of fluorescence-based microscopy that has not yet been used to visualize intracellular pathogens. Here we show the expansion of the intracellular pathogen *Chlamydia trachomatis*, enabling to differentiate its two distinct forms, catabolic active reticulate bodies (RB) and infectious elementary bodies (EB), on a conventional confocal microscope. We show that ExM enables the possibility to precisely locate chlamydial effector proteins, such as CPAF or Cdu1, within and outside of the chlamydial inclusion. Thus, we claim that ExM offers the possibility to address a broad range of questions and may be useful for further research on various intracellular pathogens.

## Introduction

Pathogen infections have been studied mainly by diffraction-limited conventional microscopy. Super-resolution microscopy methods now can provide spatial resolution that is well below the diffraction limit of light microscopy approaching virtually molecular resolution (Hell, 2007; Sauer and Heilemann, 2017). Super-resolution microscopy methods, such as stimulated emission depletion (STED) microscopy (Hell, 2007), photoactivated localization microscopy (PALM) (Betzig et al., 2006) and *direct* stochastic optical reconstruction microscopy (*d*STORM) (Heilemann et al., 2008), can be applied to biological samples and provide new and exciting views on the structural organization of cells and biomolecular assemblies.

Physical expansion of the cellular structure of interest represents an alternative approach to bypass the diffraction limit and enable “super-resolution imaging” on standard fluorescence microscopes. For this purpose, expansion microscopy (ExM) has been developed and successfully applied to visualize cellular structures with nanoscale spatial resolution using confocal laser scanning microscopy (Chen et al., 2015). The original ExM protocol attaches fluorescently labeled antibodies carrying a methacryloyl group to the protein of interest which is covalently linked into a swellable hydrogel. After degradation of native proteins by application of proteinase the sample expands uniformly ~4.5-fold in water (Chen et al., 2015). ExM has been widely used to study intracellular structures like centrioles, tissue, and to quantify RNA distributions using various protocols differing in the digestion or gelation of the sample (Gambarotto et al., 2019; Wassie et al., 2019). However, it has not yet been successfully applied to expand pathogens during infections.

*Chlamydia trachomatis* is an obligate intracellular bacterium and major human pathogen with more than 100 million infections annually. Infection causes blinding eye disease called trachoma or sexually transmitted diseases (STD), leading eventually to ectopic pregnancy and infertility. *Chlamydia* has a biphasic developmental-cycle, changing between two morphologically and physiologically distinct forms. The first phase is the electron dense, infectious elementary body (EB) with a diameter of only 0.3 μm. EBs have a reduced metabolic activity, but are able to survive the harsh extracellular conditions. Upon infection, EBs transform to the bigger and metabolically active reticulate bodies (RBs) replicating inside a vacuole, the chlamydial inclusion, via a unique polarized budding process (Abdelrahman et al., 2016). To complete the cycle RBs redifferentiate to EBs that are then released by host cell lysis or extrusion, ready to infect further cells (Hybiske and Stephens, 2007). However, distinguishing single chlamydial particles in the inclusion or in different replicating states by super-resolution fluorescence imaging remains challenging.

Here, we demonstrate the suitability of ExM to study the infection of intracellular pathogens and provide a protocol to expand the intracellular pathogen *C. trachomatis* and demonstrate its use by taking a closer look into its two distinct forms and at two important chlamydial effector proteins: CPAF and Cdu1.

## Materials and Methods

### Cell Lines and Bacteria

Human HeLa229 cells (ATCC CCL-2.1^tm^) were cultured in 10% (v/v) heat inactivated FBS (Sigma-Aldrich) RPMI1640 + GlutaMAX^tm^ medium (Gibco^tm^). They were grown in a humidified atmosphere containing 5% (v/v) CO_2_ at 37°C. In this study *C. trachomatis* serovar L2/434/Bu (ATCC VR-902B^tm^) and *C. trachomatis* mutant strains [cdu1:: Tn *bla*, CPAF (RSTE4 from Raphael Valdivia)] and the EB-RB reporter strain *Ct* mCh(GroL2) GFP(OmcAL2) (Cortina et al., 2019) were used. They were cultured and purified as previously described: *Chlamydia* were propagated in HeLa 229 cells at a multiplicity of infection (MOI) of 1 for 48 h. Afterwards the cells were detached and lysed using glass beads (3 mm, Roth). Low centrifugation supernatant (10 min at 2,000 g at 4°C) was transferred to high speed centrifugation (30 min at 30,000x g at 4°C) to pellet the bacteria. The pellet was then washed and resuspended in 1x SPG buffer (7.5% sucrose, 0.052% KH2PO4, 0.122% NaHPO4, 0.072% L-glutamate). Aliquots of the resuspended bacteria were stored at −80°C and titrated for a MOI of 1 for further experimentation. Infected cells were incubated in a humidified atmosphere with 5% (v/v) CO2 at 35°C. The cell lines as well as the *Chlamydia* used in this study were tested to be free of Mycoplasma via PCR.

### Immunostaining and ExM

For immunostaining the seeded and infected cells on cover slips were washed with 1xPBS and then fixed using 4% PFA for 30 min at RT. For the inhibition of CPAF, cells were treated with 150 μM clasto-lactacystin beta-lactone for 1 h prior to fixation according to Johnson et al. (2015). Afterwards the cells were washed three times using 1xPBS and then permeabilized with 0.2% Triton-X100 in 1x PBS for 15 min. Afterwards, the cells were blocked using (2% FCS in 1x PBS) for 1 h. The cells were then incubated in primary antibody diluted in blocking buffer for 1 h in a humid chamber. The primary antibodies used were: Anti-Cdu1 (mouse polyclonal, dilution 1:50), anti-CPAF (Provided by G. Zhong, University of Texas Health Science Center at San Antonio, mouse, dilution 1:100), anti-HSP60 (homemade, rabbit, dilution 1:200), anti-GFP (ab1218, mouse, dilution 1:200), and anti-mcherry (ab167453, rabbit, 1:500). After incubation with the primary antibody, the samples were washed 3 times in 1x PBS and then incubated in the corresponding secondary antibody diluted in blocking bother for another 1 h. The secondary antibodies used were: Alexa 488 (Thermo Fisher, A-11008, dilution 1:200) and ATTO 647N (Rockland, 610-156-121S, dilution 1:200). Afterwards the cells were washed 3 times with 1x PBS.

### Gelation and Expansion

Fixed and stained cells were treated as previously reported (Chozinski et al., 2016). After immunostaining the cells were incubated in 0.25% Glutaraldehyde (Sigma, G5882) for 10 min, washed in PBS and the glass slide then turned upside down on a drop of monomer solution [8.625% sodium acrylate, Sigma, 408220, 2.5% acrylamide, Sigma, A9926, 0.15% N,N′-methylenbisacrylamide, 2 M NaCl (Sigma, S5886) and 1x PBS] containing 0.2% freshly added ammonium persulfate (APS, Sigma, A3678) and tetramethylethylenediamine (TEMED, Sigma, T7024). The gel polymerization was performed for 1 h at room temperature in a modified reaction chamber. After gelation the glass slides were removed with tweezers and the gels were transferred in digestions buffer [50 mM Tris pH 8.0, 1 mM EDTA (Sigm, ED2P), 0.5% Triton X-100 (Thermo Fisher, 28314) and 0.8 M guanidine HCl (Sigma, 50933)], containing 8 U/ml protease K (Sigma, P4850) and digested over night at room temperature. The following day the gels were placed into excess of ddH_2_O to expand. The water was exchanged every hour until the expansion saturated. Expanded gels were chopped in small pieces (1–2 cm) and transferred in PDL-coated chambers (Merck, 734–2055). To avoid shrinking, drops of water were added on top of the gels during image acquisition.

### Microscopy

Confocal imaging was performed on an inverted microscope [Zeiss 700; 5 mW red laser (637 nm) and a 10 mW blue laser (488 nm)] using a water immersion objective (C-Apochromat, 63x 1.2 NA, Zeiss, 441777-9970). Z-stacks were processed using Imaris 8.4.1 and FIJI 1.51 n.

## Results

**Figure 1:**
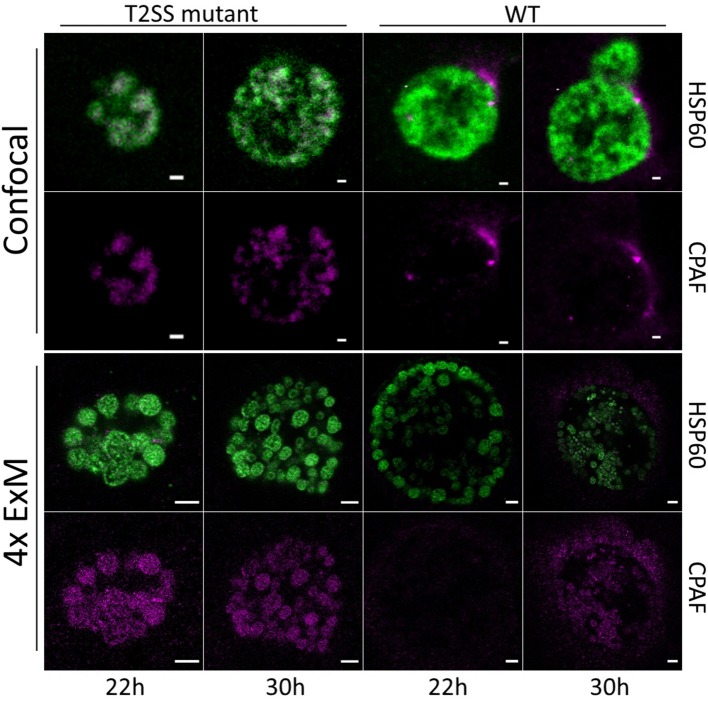
Confocal fluorescence images of *Chlamydia* infected and expanded cells. HeLa229 cells were infected for 22 and 30 h with wildtype or RSTE4 and stained for HSP60 (green, Alexa 488) and CPAF (magenta, ATTO 647N) by immunolabeling. In *C. tr* wildtype CPAF is found on individual chlamydial particles and is being secreted into the inclusion. The expression increases strongly at 30 h post infection. In the *C. tr* T2SS-mutant CPAF is not secreted and accumulates in the individual chlamydial particles. Scale bars: 1 μm for unexpanded and 5 μm for expanded images.

**Figure 2:**
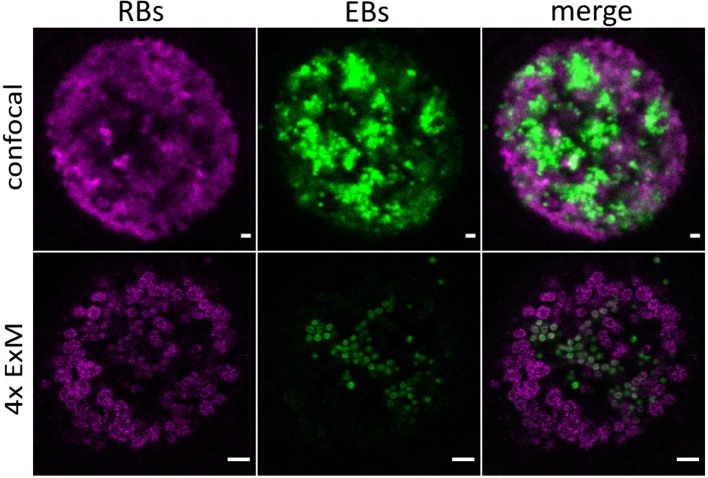
Detection of distinct chlamydial forms by expansion of infected cells. Confocal images of unexpanded and expanded HeLa229 cells infected for 30 h with *Ct* mCh(GroL2) GFP(OmcAL2) and stained for GFP (green, Alexa 488) and mCherry (magenta, ATTO 647N). ExM images show a clear difference in size of EBs and RBs. Scale bars: 1 μm for unexpanded and 5 μm for expanded images.

CPAF, chlamydial protease-like activity factor, is a chlamydial effector protein that was shown to accumulate in the inclusion of infected cells. It is secreted via a type II secretion system (T2SS) pathway. Initially, it was discovered to degrade cellular cytosolic proteins (Zhong et al., 2001). However, the localization of CPAF in the cytosol remains challenging and its function is currently under debate (Chen et al., 2012; Conrad et al., 2013; Hacker, 2014; Snavely et al., 2014; Zhong, 2014; Johnson et al., 2015). Moreover, it was shown that CPAF acts as an effector mediating the evasion of *Chlamydia* from the innate immune response as CPAF-deficient *Chlamydia* are subsequently killed by activated PMNs (Rajeeve et al., 2018). To investigate the localization of CPAF in expanded samples of HeLa229 cells, cells were infected with wildtype *Chlamydia* and a T2SS-mutant that is unable to secrete CPAF into the lumen of the inclusion (Snavely et al., 2014). After immunostaining the cells were treated with 0.25% glutaraldehyde to link the antibodies into the acrylamide gel and after polymerization digested by protease K to enable isotropic expansion. Subsequently the gels were expanded for ~4 h in ddH_2_0 to achieve a 4-fold expansion. The expansion factor was estimated by the size of EBs (0.3 μm) and RBs (0.5–0.6 μm). Isotropic expansion was determined from the size of the expanded gel and the morphology of the individual chlamydial particles. CPAF is expressed and secreted in wildtype *Chlamydia* in higher quantities at later time points of infection (Movies S1–S4). To exclude potential artifacts due to the activity of CPAF during fixation, we treated infected cells with the CPAF inhibitor clato-lactacystin beta-lactone as previously described (Johnson et al., 2015), which did not have any impact on the localization of CPAF (Figure S1). Furthermore, our data show that ExM provides the possibility to distinguish between the smaller EBs and larger RBs according to their size on a conventional microscope. EBs are more commonly found at later stages of infection (30 h) in wildtype *Chlamydia* (Figure 1; Movie S4). To confirm the distinguishability of EBs and RBs by size we made use of a recently developed *Chlamydia* strain *Ct* mCh(GroL2) GFP(OmcAL2) expressing mCherry under the control of the constitutive *groESL* operon promoter and GFP fused to the *omc* promotor (expressed in EBs; Cortina et al., 2019). Confocal images revealed the different chlamydial forms by fluorescence protein expression but differences in sizes are barely detectable. Thus, we applied ExM, which enables a clear differentiation between EBs and RBs (Figure 2; Movie S5).

**Figure 3:**
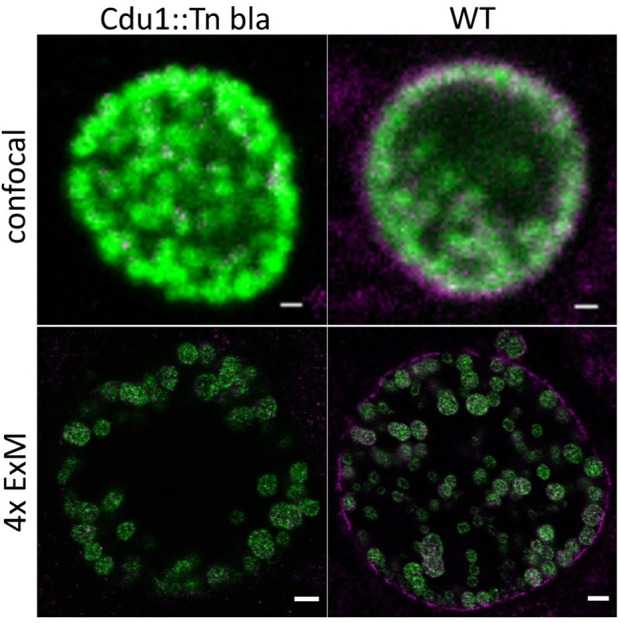
Localization of Cdu1 in the inclusion membrane by expansion of infected cells. ExM confocal images of HeLa229 cells infected for 24 h with *C. tr* WT or *C. tr* Cdu1:: Tn bla and stained for chlamydial HSP60 (green, Alexa 488) and Cdu1 (magenta, ATTO 647N). In *C.tr* wildtype Cdu1 is found in the inclusion membrane, while no Cdu1 is being detected in *C.tr* Cdu1::Tn bla mutants. Scale bars: 1 μm for unexpanded and 5 μm for expanded images.

The ubiquitin system exclusively found in eukaryotic cells is involved in a multitude of cellular functions including autophagy and cell survival as well as degradation, organellar targeting, activation, and inactivation of proteins. Several viruses and bacteria developed strategies to interfere with the host ubiquitin system. The genome of *C. trachomatis* harbors two genes encoding for deubiquitinases, ChlaDUB1 (Cdu1) and ChlaDUB2 (Cdu2). Cdu1 is a chlamydial effector deubiquitinase located at the inclusion membrane, facing the host cell cytosol with its active site (Fischer et al., 2017). The expression of Cdu1 starts around 16 h post infection and increases until the late phases of infection (Belland et al., 2003). Cdu1 has been implicated in the stabilization of IκBa and inhibition of NFkB-induced inflammatory response of the cell (Le Negrate et al., 2008) and the fragmentation of the Golgi apparatus (Pruneda et al., 2018). Moreover, Cdu1 was shown to stabilize the antiapoptotic protein Mcl-1 (Fischer et al., 2017). To investigate the localization of Cdu1 at higher spatial resolution, we expanded samples of HeLa229 cells infected with wildtype *Chlamydia* and a Cdu1::Tn bla mutant as a control (Fischer et al., 2017). Cdu1 was clearly visible in the inclusion membrane of wildtype infected cells (Figure 3; Movies S6, S7).

## Discussion

Taken together, our data demonstrate that expansion microscopy is a useful tool to study infected cells. ExM enables the visualization of nanoscale details of infection processes such as individual chlamydial particles. Furthermore, using expansion microscopy, individual chlamydial particles can be discriminated and detected as elementary and reticulate bodies due to their size by confocal fluorescence imaging (Figure 2; Movie S5). The smaller elementary bodies are more commonly found at later stages of infection (30 h) in wildtype *Chlamydia* (Figure 1; Movie S4). Hence, it can be used to address a variety of important infection-relevant questions without the need to establish elaborate super-resolution microscopy methods. The sensitivity of ExM was comparable to traditional LSM imaging but we had to overcome dye fading problems during the gelation procedure. This could be achieved by using the two extremely stable dyes ATTO 647N and Alexa 488 to minimize fading effects. Interestingly, some ExM protocols allow additional post-labeling and even accessing new epitopes generated during the denaturation step (Gambarotto et al., 2019).

An interesting observation was the association of CPAF with chlamydial particles despite the secretion into the inclusion lumen at later time points. At this stage we can however not exclude, that the signal originates from intracellular, non-secreted CPAF. There are now several reports that show that host proteins are cleaved or degraded by CPAF outside of the *Chlamydia*-infected cell (Tang et al., 2015; Yang et al., 2016; Rajeeve et al., 2018). It is assumed that the enzymatically active form of CPAF is released upon cell rupture that cleaves host proteins in the extracellular milieu. It is also possible, that EBs carry active CPAF on their surface. This would suggest that CPAF activity remains associated even with released EBs which would support a function of CPAF in protecting EBs from innate immune defense such as the activity of antimicrobial peptides like cathelicidin LL-37 (Tang et al., 2015), complement (Yang et al., 2016), or neutrophil attack (Rajeeve et al., 2018).

Our results indicate that ExM is generally suitable to study bacterial and maybe even infections with larger viruses like influenza, herpes, or HIV down to a particle size of ~65 nm. As demonstrated by others, tissue of mouse models and even patients can be expanded and imaged in multicolor 3D ExM (Wassie et al., 2019). Accordingly, ExM offers a simple and extremely versatile method to study infections on a confocal microscope close to the resolution of sophisticated super-resolution microscopes.

## Data Availability

The raw data supporting the conclusions of this manuscript will be made available by the authors, without undue reservation, to any qualified researcher.

## Author Contributions

TK, RG, MS, and TR conceived the study, wrote the manuscript, and edited the manuscript. TK and RG performed the experiments and analyzed the data. TR and MS supervised the study.

### Conflict of Interest Statement

The authors declare that the research was conducted in the absence of any commercial or financial relationships that could be construed as a potential conflict of interest.

## Supplementary Material

Movie S1 Confocal fluorescence z-stacks of T2SS mutant *Chlamydia* infected (22 h) and expanded cells. Hela229 cells were infected for 22 h with RSTE4 and stained for HSP60 (green, Alexa 488) and CPAF (magenta, ATTO 647N) by immunolabeling. CPAF is expressed but not secreted.

Movie S2 Confocal fluorescence z-stacks of T2SS mutant *Chlamydia* infected (30 h) and expanded cells. Hela229 cells were infected for 30 h with RSTE4 and stained for HSP60 (green, Alexa 488) and CPAF (magenta, ATTO 647N) by immunolabeling. CPAF is expressed but not secreted.

Movie S3 Confocal fluorescence z-stacks of wildtype *Chlamydia* infected (22 h) and expanded cells. Hela229 cells were infected for 22 h with wildtype *C. tr* and stained for HSP60 (green, Alexa 488) and CPAF (magenta, ATTO 647N) by immunolabeling. CPAF is expressed and secreted.

Movie S4 Confocal fluorescence z-stacks of wildtype *Chlamydia* infected (30 h) and expanded cells. Hela229 cells were infected for 30 h with wildtype *C. tr* and stained for HSP60 (green, Alexa 488) and CPAF (magenta, Atto 647N) by immunolabeling. CPAF is strongly expressed, secreted, and accumulates in the individual chlamydial particles.

Movie S5 Confocal fluorescence z-stacks of *Chlamydia* infected and expanded cells. Hela229 cells were infected for 30 h with *Ct* mCh(GroL2) GFP(OmcAL2) and stained for GFP (green, Alexa 488) and mCherry (magenta, ATTO 647N) by immunolabeling.

Movie S6 Confocal fluorescence z-stacks of Cdu1 mutant *Chlamydia* infected (24 h) and expanded cells. Hela229 cells were infected for 24 h with *C. tr* Cdu1:: Tn bla and stained for HSP60 (green, Alexa 488) and Cdu1 (magenta, ATTO 647N) by immunolabeling. Cdu1 is not expressed.

Movie S7 Confocal fluorescence z-stacks of wildtype *Chlamydia* infected (24 h) and expanded cells. Hela229 cells were infected for 24 h with wildtype *C. tr* and stained for HSP60 (green, Alexa 488) and Cdu1 (magenta, ATTO 647N) by immunolabeling. Cdu1 is expressed and localizes to the inclusion membrane.

Figure S1 Confocal fluorescence images of *Chlamydia* infected, clasto-lactacystin beta-lactone treated, and expanded cells. HeLa229 cells were infected for 30 h with wildtype *Chlamydia*. Prior to fixation the cells were treated with 150 μM clasto-lactacytin beta-lactone for 1 h. The samples were stained for HSP60 (green, Alexa 488) and CPAF (magenta, ATTO 647N). Scale bars: 1 μm for unexpanded and 5 μm for expanded images.
