# Supplementary figures and images for: Detection of Chlamydia Developmental Forms and Secreted Effectors by Expansion Microscopy
Source: Front Cell Infect Microbiol. 2019 Aug 9;9:276. doi: 10.3389/fcimb.2019.00276 (PMC6695470; doi:10.3389/fcimb.2019.00276)

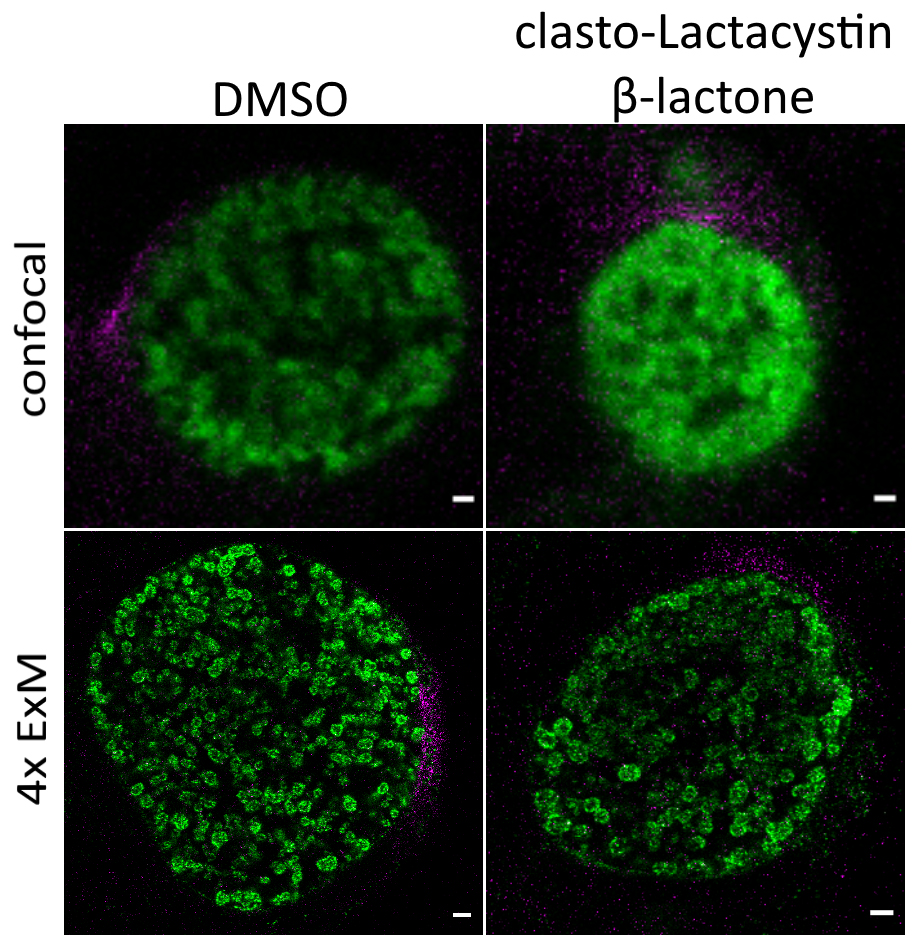

Supplement: Figure S1 — Confocal fluorescence images of Chlamydia infected, clasto-lactacystin beta-lactone treated, and expanded cells. HeLa229 cells were infected for 30 h with wildtype Chlamydia. Prior to fixation the cells were treated with 150 μM clasto-lactacytin beta-lactone for 1 h. The samples were stained for HSP60 (green, Alexa 488) and CPAF (magenta, ATTO 647N). Scale bars: 1 μm for unexpanded and 5 μm for expanded images. [file Image_1.JPEG]
